# Elementary Chemistry, Theoretical and Practical

**Published:** 1845-09

**Authors:** 


*Elementary Chemistry, Theoretical and Practical,* By George Fownes, Ph. D., Chemical Lecturer in the Middlesex Hospital Medical School, &c., with numerous Illustrations. Edited with Additions, by Robert Bridges, M. D. Professor of General and Pharmaceutical Chemistry in the College of Pharmacy, &c., &c. Philad. Lea & Blanchard, 1845. pp. 460, 12 mo. (from the Publishers.)

This work is intended as a manual for students, and is, in our opinion, one of the best, if not the very best, for this purpose, that has ever come under our revision. It is written in a pleasing and lucid style, and although truly elementary, is by no means superficial. The illustrations are sufficiently numerous and much to the purpose. In the English edition the science was brought up to the date of publication, and the editor of the American edition has performed his part of the task with his usual ability. The book, as presented to the American student, is a complete epitome of the science, sufficiently voluminous and not too minute. A proper proportion of the work is devoted to Organic Chemistry; and this part of the science, although much more difficult to render clear to the beginner than the Inorganic, has been brought down to the comprehension of the merest tyro. The mechanical execution of the work is good. Considering the work in all points excellent, and adapted to the wants of the student, we will take pleasure in recommending it to our class as a text book, and to students generally as a manual.—Ed.

